# Soil characteristics and allometric models for biometric characteristics and nutrient amounts for high yielding “Bolaina” (*Guazuma crinita*) trees

**DOI:** 10.1038/s41598-024-52790-1

**Published:** 2024-01-30

**Authors:** C. O. Arévalo-Hernández, E. Arévalo-Gardini, J. A. Correa V., J. O. Souza Júnior, J. C. L. Neves

**Affiliations:** 1 Department of Soils, Instituto de Cultivos Tropicales (ICT), Tarapoto, Peru; 2 Professional School of Agronomic Engineering, Universidad Nacional Autonoma de Alto Amazonas (UNAAA), Yurimaguas, Peru; 3 Department of Agricultural and Environmental Sciences, Universidade Estadual de Santa Cruz (UESC), Ilhéus, Brazil; 4 Department of Soils, Universidade Federal de Viçosa (UFV), Viçosa, Brazil

**Keywords:** Plant physiology, Forestry

## Abstract

The Peruvian amazon is very diverse in native forestry species, the *Guazuma crinita* “Bolaina” being one of the most planted species in the country; however, little or no information about soil requirements and nutrient demands is known. The objective of this work was to assess the general conditions of soil fertility, biomass and macro- and micronutrient amounts in high-productivity *Guazuma crinita* plantations. Fields of high yielding Bolaina of different ages (1–10 years) were sampled in two regions. Soil and plant samples were collected in each field and biometric measurements of fresh weight, diameter at breast height and height were performed. For soil and plant analysis, both macro- (N, P, K, Ca, Mg, S) and micronutrients (B, Cu, Fe, Mn, Zn) were determined. Finally, allometric equations were constructed for biometric and nutrient amounts. This study is the first to assess and model macro- and micronutrient amounts in the productive cycle in this species, which grows in fertile soils. In the case of biometric equations, the logarithmic and logistic models performed better. For nutrient amounts, this species followed a pattern of Ca > N > K > P > S > Mg for macronutrients and Fe > B > Mn > Zn > Cu for micronutrients. The best prediction models for nutrients were the square root and logistic models.

## Introduction

The Peruvian Amazon covers the largest area in Peru, representing nearly 60% of the total territory. The Amazon is characterized by high diversity in both flora and fauna, being one of the most diverse ecosystems worldwide^1^. This region is characterized by a multitude of native trees that have several purposes, such as conserving biodiversity, carbon stocks, water cycling, traditional medicine and wood^2,3^. Recently the Peruvian government has focused on promoting forestry plantations and exploitation by assigning resources in terms of credits, funds for projects and loans for each purpose, all of which were considered in the “Plan for Promoting Commercial Forestry Plantations 2021–2050”^4^. However, this promotion for forestry business has been focused on some species with some level of knowledge about their management and cultivation, such as *Eucalyptus* sp., “Capirona” (*Calycophyllum spruceanum*), “Cedro” (*Cedrela odorata*), *Pinus* sp. and “Bolaina” (*Guazuma crinita*). The latter has been greatly promoted by the government in the last decade because of its rapid growth, feasibility of work and a relatively new demand on the market based on white wood^5^.

In general, forestry plantations in Peru are small in comparison to the areas dedicated to agriculture and of the 117,495.59 ha of plantations in 2022 the areas dedicated to *Guazuma crinita* amounted to 15,109.46 ha, representing nearly 12.86% of forestry plantations in the country, and it is also the most planted species in Peru^6^. Even with its high importance, research on this species has focused mostly on genetics^7–9^, developing allometric models^10,11^, carbon storage^12^, propagation^13^, wood characteristics^14^ or management^15,16^, with little known about soil fertility, nutrition requirements or fertilization management in this species, which represent very important issues for future commercial plantations.

Nutrition management is a particularly important topic in the case of forestry plantations, even though in the past it was considered that trees could grow without inputs and in very infertile soils^17^. Nowadays, the use of fertilizers and soil amendments is known to improve growth, development and wood quality^18,19^. Also, temperature regimes in the tropics are very high (in the coldest month, > 18 °C) in comparison to other latitudes, which benefits trees by operating at its optimum condition, allowing them to grow faster and making nutrition requirements more critical when water supply is not limiting^20,21^.

In the case of tropical native species, an extensive review by^17^ summarizes that most species in the tropics demand nutrients in the order N > K > Ca > Mg > P and that concentrations of nutrients on the litter are higher in Ca and N and higher in deciduous species in comparison to evergreen species. In the case of *Guazuma crinita*, few studies on nutrition or fertilizer requirements exist; most are related to the response to organic and inorganic fertilizers in greenhouse^22^ and field conditions^23–25^, where all the authors found positive responses for biometric measurements (height, diameter, dry weight, foliar area, root growth) when fertilizers or amendments were used. From these studies, only^22,24^ analyzed foliar concentration of nutrients, but only for N, P and K, where they reported concentrations of nutrients in the order N > K > P.

Soil fertility is important for ecology restoration in tropical ecosystems because soil conditions may limit the establishment and growth of new forestry trees in degraded areas. Therefore, general knowledge of basic soil conditions may improve the selection of areas for reforestation of tree species^17,26^. General nutrition requirements are important for establishing fertilization programs in commercial crops worldwide to efficiently apply fertilizer and avoid excessive doses that can cause serious problems in the environment, such as salinity, contamination of groundwater, eutrophication, etc.^27^.

Therefore, taking into consideration the need for basic information related to the nutrition demands of *Guazuma crinita*, we hypothesized that high productivity stands of this species may reflect adequate conditions in terms of soil fertility, growth and nutrition in order to build prediction models that can be used for improving management in this important native tree. Therefore, this work had the following objectives: to assess the general conditions of soil fertility in high-productivity *Guazuma crinita* plantations; to explore the accumulation of biomass and nutrients (macro- and micronutrients) in different ages of high-productivity *Guazuma crinita* plantations; and to develop allometric models for biomass and nutrient uptake in *Guazuma crinita*.

## Results

### Soil characteristics of high-yield stands

Physical and chemical soil characteristics for the San Martin (SM) and Ucayali (UC) regions are presented in Table 1. With regard to physical characteristics, soils in SM had more sand and less silt compared to UC; no significant differences were observed (*P* > 0.05) for clay but the silt/clay ratio is higher in UC (1.48) than in SM (0.78), reflecting younger soils in the case of UC. In relation to chemical characteristics, soils from the collected areas had similar pH values and no significant differences (*P* > 0.05) were reported; the pH was around neutral (pH 7.0), indicating that even though this species had emerged from the Amazon region it may have a preference for neutral soils. Also, in the case of organic matter (OM) in the soil, no significant differences (*P* > 0.05) were observed between the regions and values were above the normal levels found in tropical soils (OM > 30 g kg^−1^), which may be related to the high C values required for this species. For P and S, significant differences were indicated, where the SM region had higher values than the UC region. In the case of exchangeable bases (Ca, Mg and K), significant differences (*P* ≤ 0.05) were observed only for K, where the SM region had higher values (6.2 mmol_c_ kg^−1^) in comparison to UC (4.0 mmol_c_ kg^−1^). In the case of available micronutrients (B, Cu, Fe, Mn and Zn), only B showed significant differences (*P* ≤ 0.05), with the SM region reporting higher values (0.15 mg kg^−1^) in comparison to UC (0.02 mg kg^−1^); however, both regions showed low available concentrations of this element.

**Table 1:** Physical (sand, silt and clay proportions) and chemical [pH, CaCO_3_, organic matter (OM), Ca, Mg, K, P, S, B, Cu, Fe, Mn and Zn] characteristics of soils in the San Martin (SM) and Ucayali (UC) regions.

Region	Sand	Silt	Clay	pH	CaCO_3_	OM	Ca	Mg	K	P	S	B	Cu	Fe	Mn	Zn
	g kg^−1^				g kg^−1^		mmol_c_ kg^−1^			mg kg^−1^						
SM	378 ± 116	273 ± 67	349 ± 68	7.00 ± 0.56	13.8 ± 12.6	42.2 ± 14.7	244 ± 104	21 ± 5	6.2 ± 3.7	18.1 ± 10.4	18.9 ± 14.6	0.15 ± 0.10	0.95 ± 0.95	28.3 ± 21.8	26.2 ± 14.8	3.3 ± 2.2
UC	243 ± 120	45.1 ± 190	30.6 ± 121	6.77 ± 0.41	11.5 ± 17.3	38.8 ± 10.2	254 ± 73	23 ± 6	4.0 ± 1.3	8.4 ± 3.7	7.0 ± 7.9	0.02 ± 0.02	0.94 ± 0.52	38.4 ± 13.9	28.8 ± 12.3	2.5 ± 1.6
*P*	*	*	ns	ns	ns	ns	ns	ns	*	*	**	*	ns	ns	ns	ns

Granulometric analysis (Bouyoucos method), pH (potentiometer 1:2.5), CaCO_3_ (calcimeter), OM (Walkley and Black), P (0.5 mol L^−1^ NaHCO_3_, pH 8.5), Ca, Mg, K (1.0 mol L^−1^ NH_4_OAc), S [500 mg L^−1^ P with Ca(H_2_PO_4_) 2H_2_O + 2.0 mol L^−1^ CH_3_COOH], B (hot water) and Cu, Fe, Mn and Zn (diethylenetriaminepentaacetic acid: DTPA).

*ns* not significant.

*significant at 0.05.

**significant at 0.01.

### Biometric measurements

The mean biometric measurements per selected age are presented in Table ESI-1 (see Supplementary Information). In the case of biometric measurements, allometric models were developed using data from both regions (SM and UC) as non-significant differences were observed (*P* > 0.05) between the biometric measurements in these regions and, in this way, the constructed models can be improved with more data. The tested models, formulae, Akaike information criterion (AIC) and root-mean-square error (RMSE) are presented in Table 2 [commercial height (CH) and diameter at breast height (DBH)] and Table 3 (dry weight). For CH and DBH estimation, the best model was the square root model (Model 2), with *R*^2^ values of 0.91 and 0.79 for CH and DBH, respectively.

**Table 2:** Allometric model evaluation with the Akaike information criterion (AIC) and root-mean-square error (RMSE) for commercial height (CH) and diameter at breast height (DBH) of “Bolaina” (*Guazuma crinita*) trees, based on DBH and age, respectively, from 1 to 10 years in the sampled areas of the San Martin and Ucayali regions.

Models	Commercial height (m)		Diameter at breast height (cm)	
	AIC	RMSE	AIC	RMSE
1	26.68	0.33	− 41.73	0.12
2	**− 33.76**	**0.13**	− **42.03**	**0.12**
7	20.92	0.30	− 36.73	0.13
8	15.53	0.28	− 34.76	0.14
9	− 19.15	0.16	− 41.64	0.12
10	− 13.30	0.18	− 41.73	0.12
Best model formula*	$Ln \left(DBH\right)=2.4259+0.1012 *{Years}^{0.5}-0.0632* Years$, *R*^2^ = 0.79	$Ln \left(DBH\right)=2.4259+0.1012 *{Years}^{0.5}-0.0632* Years$, *R*^2^ = 0.79	$Ln \left(DBH\right)=2.4259+0.1012 *{Years}^{0.5}-0.0632* Years$, *R*^2^ = 0.79	$Ln \left(DBH\right)=2.4259+0.1012 *{Years}^{0.5}-0.0632* Years$, *R*^2^ = 0.79

*The selected models were significant by the *F*-test at 0.05. Values in bold indicate the best model, with the lower AIC and RMSE values.

**Table 3:** Allometric model evaluation with the Akaike information criterion (AIC) and root-mean-square error (RMSE) for trunk, branches, leaves and total dry weight (*Dw;* in kg) of “Bolaina” (*Guazuma crinita*) trees from 1 to 10 years in the sampled areas of the San Martin and Ucayali regions.

Models	Trunk		Branches		Leaves		Total	
	AIC	RMSE	AIC	RMSE	AIC	RMSE	AIC	RMSE
1	34.04	0.36	67.78	0.58	99.16	0.86	12.28	0.26
2	− 21.23	0.16	68.55	0.57	100.83	0.87	-27.63	0.15
3	− **32.68**	**0.14**	70.69	0.61	102.36	0.89	-19.69	0.17
4	− 28.76	0.15	68.76	0.65	102.76	0.89	-22.51	0.16
5	19.38	0.14	69.75	0.55	101.59	0.88	2.78	0.15
6	− 28.37	0.14	**65.03**	**0.55**	100.54	0.87	-23.59	0.15
7	24.95	0.32	68.38	0.59	**98.89**	**0.86**	-0.04	0.22
8	11.45	0.26	68.60	0.59	100.01	0.87	-10.89	0.19
9	− 22.33	0.16	72.33	0.57	101.33	0.88	**-28.46**	**0.14**
10	− 16.72	0.17	86.72	0.57	108.72	0.94	-26.20	0.15
Best model formula	$Ln\left(Dw\right)=-370.18+55.52*\mathrm{log }({{\text{DBH}}}^{2}*H)$, *R*^2^ = 0.96		$Ln(Dw)= 0.0008*{{\text{DBH}}}^{2.9435}$, *R*^2^ = 0.62		$Ln(Dw)= 1.1165*{{\text{DBH}}}^{1.4163}*{H}^{-0.5786}$, *R*^2^ = 0.27		$Ln \left(Dw\right)=\frac{5.6277}{1+{e}^{1.5581-0.1675*DBH} },$ *R*^2^ = 0.93	

*The selected models were significant by the *F*-test at 0.05. Values in bold indicate the best model, with the lower AIC and RMSE values.

In the case of dry weight estimation (Table 3), poor predictive models were constructed for leaves and branches. However, trunk and total weight had good prediction estimations, with *R*^2^ values of 0.96 and 0.93, respectively, and the best predictive models were the logarithmic model (Model 3) and the logistic model (Model 9), respectively. In the case of leaves, the best model was the power model (Model 7), with *R*^2^ = 0.62, whereas for branches it was the power model (Model 6), with *R*^2^ = 0.25. In general, the highest proportion of biomass in these trees is represented by the trunk, with a lesser proportion for leaves and branches; with increasing age this relation is higher, accounting for 56.80% of the total biomass of the tree in the first year to 77.70% at 10 years. In the case of leaves and branches, this relation accounts for 5.07% and 38.13% in the first year to 10.11% and 12.19% at 10 years, respectively.

### Macronutrient amounts

**Figure 1:**
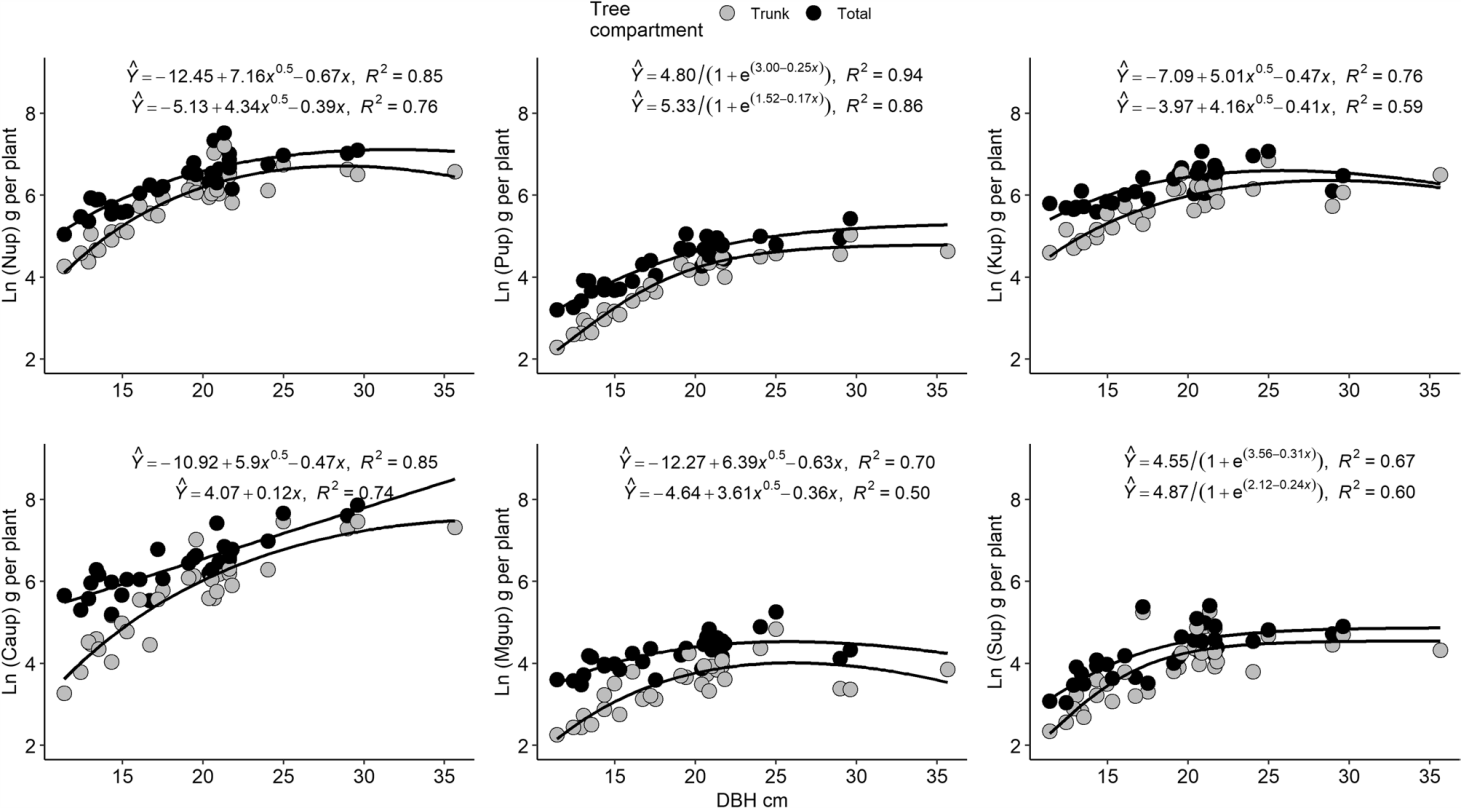
Allometric equations for macronutrient (N, P, K, Ca, Mg, S) amounts (in g per plant) in relation to diameter at breast height (DBH) in *Guazuma crinita* “Bolaina blanca” plants. The square root model was used for N, K, Ca and Mg and the logistic model for P and S.

The mean concentrations and amounts of each macronutrient according to age are presented in Table ESI-2 (see Supplementary Information). In the case of nutrient amounts, the tested equations in terms of AIC and RMSE for each nutrient are presented in Table ESI-3 and the best-selected allometric equations are presented in Fig. 1. For nutrient amounts, the square root model obtained the best fit for trunk and total amounts of N, K, Ca and Mg whereas the logistic model obtained better results for P and S. In general, at a younger age (DBH < 15 cm), the accumulation of macronutrients was as follows: N > Ca > K > P > S > Mg. However, at a later age or higher DBH, macronutrient amounts followed the order Ca > N > K > P > S > Mg.

For nutritional demand, the best models for estimating the absorption of nutrients in *Guazuma* were used. To calculate the optimal nutrient demand in the *Guazuma crinita* productive cycle, some considerations were made. For the square root equation, a derivative of the equation was used to calculate the maximum DBH, whereas for the exponential and logarithmic equations the asymptote was considered as this value. However, in all equations, a value of 3 Baule units (87.5%) was used in relation to the maximum DBH to estimate the optimal demand rate and avoid excessive calculations for fertilizer programs in this species. The overall macronutrient demands (trunk and total) are summarized in Table 4. In the case of Ca, the total estimation was by taking into consideration the maximum value obtained by the square root model and then using the linear formula for total Ca uptake estimation.

**Table 4:** Macronutrient (N, P, K, Ca, Mg, S) demands in “Bolaina” (*Guazuma crinita*) over the productive cycle, considering 400 plants per hectare.

N		P		K		Ca		Mg		S	
Trunk	Total	Trunk	Total	Trunk	Total	Trunk	Total	Trunk	Total	Trunk	Total
g per plant											
757.1	1163.7	66.5	106.2	545.0	702.3	1705.3	4093.1	51.6	89.1	53.4	71.0
kg ha^−1^											
302.8	465.5	26.6	42.5	218.0	280.9	682.1	1473.5	20.6	35.6	21.4	28.4

### Micronutrient amounts

**Figure 2:**
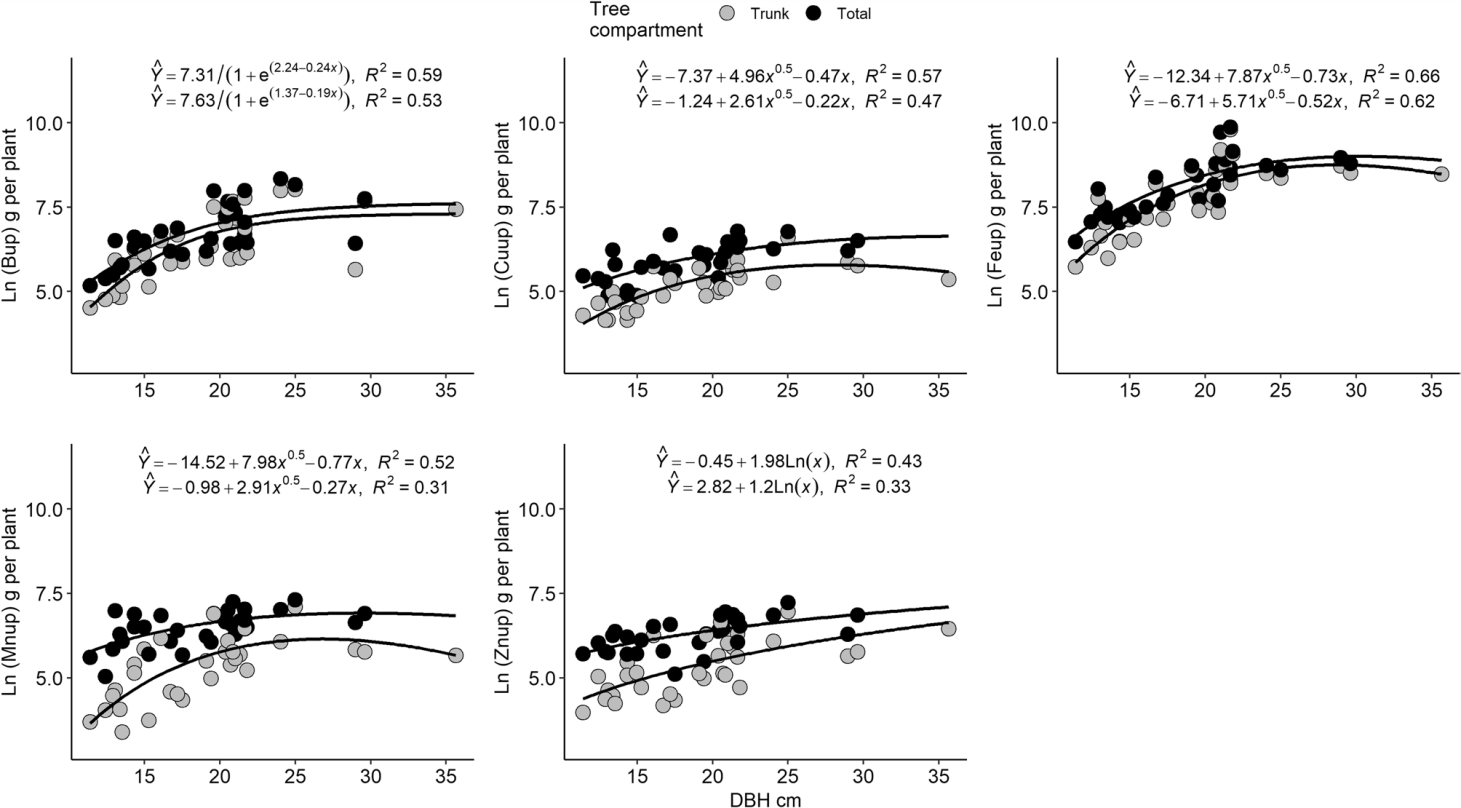
Allometric equations for micronutrient (B, Cu, Fe, Mn, Zn) amounts (in g per plant) in relation to diameter at breast height (DBH) in *Guazuma crinita* “Bolaina blanca” plants. The logistic model was used for B, the square root model for Cu, Fe and Mn and the logarithmic model for Zn.

The mean concentrations and amounts of each micronutrient according to age are presented in Table ESI-2 (see Supplementary Information). In the case of micronutrient amounts, the tested equations for each nutrient are presented in Table ESI-4 and the best-selected allometric equations are presented in Fig. 2. For nutrient amounts, the square root model obtained the best fit for trunk and total amount of Cu, Fe and Mn whereas for B it was the logistic model and for Zn it was the power model. In general, micronutrient amounts were observed in the order Fe > B > Mn > Zn > Cu. In this case, there were no variations in the order of absorption of these nutrients with higher DBH and age. With regard to the models, the prediction was lower in comparison to macronutrients (*R*^2^ > 0.60); this could be related to the higher variation in micronutrient amounts and the spatial variability normally observed for this type of variable in research.

As in the case of macronutrients, the micronutrient demands were calculated by taking into consideration the same assumptions. The results of the micronutrient demands for *Guazuma crinita* are presented in Table 5, expressed in mg per tree and g ha^-1^.

**Table 5:** Micronutrient (B, Cu, Fe, Mn, Zn) demands in “Bolaina” (*Guazuma crinita*) over the productive cycle, considering 400 plants per hectare.

B		Cu		Fe		Mn		Zn	
Trunk	Total	Trunk	Total	Trunk	Total	Trunk	Total	Trunk	Total
mg per plant									
599.9	793.0	309.0	737.9	5792.2	7630.3	429.8	775.5	240.0	610.9
g ha^−1^									
240.0	317.2	123.6	295.2	2316.9	3052.1	171.9	310.2	96.0	244.4

## Discussion

### Soils in “Bolaina” stands

In terms of the physical attributes of soil, the values of particle size analysis (sand, silt and clay) indicate that SM soils may have higher water infiltration and drainage, which can reduce water accumulation and prevent root diseases and anoxia conditions while enhancing growth^28^. Also, the higher concentration of silt in UC soils may indicate younger soil (in terms of geologic time) and higher quartz concentrations. Higher values of silt can be negatively correlated to growth in *Eucalyptus grandis*^29^. Furthermore, the silt/clay ratio for these soils was higher, which may be related to higher tree mortality in some tropical species^30^. With regard to clay, the values were higher than 250 g kg^−1^ in both regions and, as specified by, Soong et al.^31^ this may be related to lesss leaching of nutrients, which can improve overall nutrient stocks and tree growth. These observations indicate that SM-sampled soils present better conditions for *Guazuma crinita* growth. Nevertheless, even though the physical properties of soil are important for the root development of trees, its chemical attributes, especially nutrient availability, are also key players in terms of overall tree survival and growth^31,32^. In general, all the chemical attributes of soil were at a good level for the growth of any plant, with the exception of B that was found at a low level (< 0.2 mg kg^−1^); also, specifically for UC, P was at a low level (< 10 mg kg^−1^) and S at a medium level (< 20 mg kg^−1^), according to Horneck et al.^33^. These chemical results in both sampled sites partly explain the high growth in these areas, since all collected plots were attributed as high productivity plantations. In general, forestry species require low levels of P, S and B in comparison to K and Ca^17^. In particular, K is generally scarce in tropical soils due to the dominance of ultisols and oxisols^34^. However, in these plantations this element was not limited, a fact that can be associated with the less weathered soils present in these areas. With regard to Ca, this element is especially important in teak plantations^35,36^, this species being one of the main exceptions to this rule.

### Biometric measurements in “Bolaina”

In general, there is scant research regarding allometric models for estimating biomass in this species, with the exception of the work of Revilla-Chávez et al.^11^. However, the latter has only considered plants of 31 months old, with a wide range of biometric measurements to obtain *R*^2^ of the same magnitude as this work. Our research is the first to consider a wide range of age (1–10 years) for dry weight estimation in this species, making it possible for general estimations of biomass and possibly also for carbon stock estimations in these types of plantations. Also, research on allometric models in this species has focused more on the determination of commercial volumes of wood rather than dry weight^10,11,37^, so the literature is very scarce. In general, the first equations. (1–4) are commonly used in the literature for estimating height, DBH and weight, as shown by the work of Huang et al.^38^ & Vorster et al.^39^. However, the logarithmic and logistic models have shown very good results for estimating all the variables, especially for trunk and total weight in *Guazuma crinita*, indicating that their use may be interesting in further research with this species.

### Macro- and micronutrient amounts in “Bolaina”

In the case of accumulation of macronutrients in other tropical cultivated species, teak presented a similar trend to *Guazuma*: at younger ages (5 years), N > Ca > K > P > Mg > S; at later ages (10 years), Ca > N > K > Mg > P > S^40,41^. For six different *Eucalyptus* (age 3.5 years) genotypes the order was Ca > N > K > Mg > S > P at age 3.5 years^42^ but in Europe at 18 years it was N > Ca > K > Mg > P^43^, so a shift of Ca for N demand was observed. For *Larix* the order was N > Ca > K > P > Mg^44^ but for *Pinus radiata* at 35 years it was N > K > Ca > Mg > P^43^. *Guazuma* reported a high demand for Ca that can only be compared with teak; although other tree species accumulated Ca it was not on the scale of *Guazuma* and N prevailed as the nutrient in the most demand for this species.

All nutrients are important for plant growth and development but in general N and K are the most in demand, as was observed for *Guazuna* in the early years. Nitrogen is a key element for plant metabolism and is the main component of many organic compounds, such as amino acids, proteins, enzymes, etc.^45,46^. Also, the absence of this element can significantly diminish tree growth and development^36,47^, making it necessary to apply fertilizers in order to combat this limitation. On the other hand, K^+^ is a dominant cation in plant cells; it does not have a structural role as N does, but it features in several metabolic functions such as stomatal regulation, stress alleviation, water economy, enzymatic and photosynthetic activity^48^. However, in this species the dominance of another element was observed: the absorption of Ca was superior in comparison to N, which decreased exponentially with passing years and higher DBH. Cultivated soils with this species presented high Ca, and sometimes also CaCO_3_ (in low concentrations of < 2%), which could partly explain these higher concentrations. However, some forest species have been reported as high Ca-demanding species, such as teak (*Tectona grandis*), caoba (*Swietenia macrophylla*), *Pinus patula* and others^17,41^. Calcium is an important nutrient in plants for their growth and development because it forms important structural molecules, such as the cell wall and membranes^49^, and participates actively in metabolism as a secondary messenger, the oscillatory concentrations in cytosolic Ca^2+^ being the signal for responses to abiotic and biotic stress^49–51^ and its sufficiency in tropical woody plants such as *Guazuma* being important for its survival and establishment in this highly competitive environment.

In general, the literature related to micronutrient amounts in forestry is scarce in comparison to macronutrients. Nevertheless, the accumulation of micronutrients for teak (*Tectona grandis*) was in the order Fe > Zn > Mn > B > Cu^40^, for *Eucalyptus* it was Mn > Fe > B > Zn > Cu^42,43^, for *Pinus* sp. it was Mn > Fe > Zn > Cu^43,52^ for *Pinus pinaster* it was Mn > Fe > Zn > B > Cu^53^ and for *Larix* it was Mn > Fe > Zn > Cu^44^. The main difference for *Guazuma* in comparison to other tree species is the relatively high requirement for B, this being the second most demanded micronutrient throughout its production cycle.

Micronutrients are very important for plant development and are required in lesser amounts, with both Fe and Mn generally being the most in-demand micronutrients for all plants^54^; these elements are very important and can be very scarce in neutral to alkaline soils (as the sampled soils) because their availability decays drastically at high pH. Iron is one of the most abundant elements on Earth and in plants it participates in photosynthesis, respiration and, with its high reactivity, helps to transport and balance oxygen^55,56^. On the other hand, Mn is important for its functions as an enzyme cofactor in numerous enzymes, such as arginase, pyruvate carboxylase and Mn superoxide dismutase, among others^57^. However, it can also act to prevent biotic stress, hormone signaling and detoxification of reactive oxygen species^58^. In general, in Amazon soils, both Fe and Mn occur in adequate to high concentrations; however, B is generally scarce^34,59^. Boron has a structural role in the cell wall and plasma membrane and plays an important role in carbohydrate metabolism, bloom, fructification and hormonal activity; a deficiency in B can diminish wood quality^60^. Furthermore, woody trees use their reserves of B to overcome deficiency and this factor may be one of the most important when estimating the requirement for B^60,61^. In addition, B management is difficult due the narrow limit between deficiency and toxicity in plants; however, in the case of woody species, their ability to store this element may help to reduce the effect of high B application.

## Conclusion

“Bolaina” (*Guazuma crinita*) is a very important forest tree in the Peruvian Amazon, being the most planted tree in the country, and has interesting advantages such as rapid growth and its use for wood and construction. This research collected samples in different high-yielding *Guazuma* plantations in order to explore the general soil conditions and construct allometric equations for biometric variables and nutrient amounts. Overall, it was observed that this species grows in fertile soils. In the case of biometric equations, the logarithmic and logistic models performed better. For nutrient amounts, this species followed a pattern of Ca > N > K > P > S > Mg for macronutrients and Fe > B > Mn > Zn > Cu for micronutrients. The best models for prediction were the square root model (N, K, Ca, Mg, Cu, Fe, Mn), logistic model (P, S, B) and logarithmic model (Zn). This study is the first to assess and model macro- and micronutrient amounts in the productive cycle of this species and the high Ca accumulation in relation to N needs to be researched further by exploring the physiological, anatomical or molecular drivers that explain this behavior.

## Materials and methods

### Location

The study was located at three regions in Peru: San Martin, Huánuco and Ucayali. The climates of the collected areas are similar, with temperatures of 21–33 °C and precipitations higher than 1400 mm per year, classified as Am^62^.

The plantations selected were trees of seminal origin and the data collection places in the San Martin, Ucayali and Huanuco regions are presented in Fig. ESI-1 (see Supplementary Information); San Alejandro, Curimana, Puerto Inca, Pajarillo, Huicungo and Juanjui. Nevertheless, as there was only one data collection site in Huanuco the data for analysis were combined with the Ucayali data due to the similarities in environmental conditions.

### Soil and plant sampling

“Bolaina” trees of 1, 3, 5, 7 and 10 years were selected for study. Plots were selected according to their productivity based on the highest forest volume over time; as no reference exists relating high, medium or low productivity, the highest values were used. Five trees were selected based on the highest circumference in each plot. In the case of plant sampling, the procedures are according to institutional guidelines and the Peruvian national legislation (Law 29,763), which does not require permission to work with this species because the samples were collected from commercial plantations.

The selected specimens were harvested and cut in different pieces for biometric assessment (see Section “Macronutrient amounts”): trunk, branches and leaves.

For plant sampling, three circular sections from the trunk (base, middle and top) were collected; for branches and leaves, samples of 2 kg and 1 kg were sampled, respectively.

For soil sampling, four samples of 250 g were taken at 0.20 m depth, in the north, south, east and west of the tree at 20 cm distance from the trunk base. Soil sampling was performed for each tree sacrificed in the study. All the samples were stored in boxes at a temperature of 4 °C and then sent to the Instituto de Cultivos Tropicales laboratory for soil and plant analysis.

### Determination of biometric variables

The main variables determined were the height, diameter and dry weight of the trunk, branches and leaves.

With regard to height, the trees were measured in meters (m) after they were sacrificed. Circumference was measured at a height of 1.30 m and diameter was estimated by dividing the circumference by the value of pi.

Fresh weight was measured in the field and sections of different parts of the tree were preserved at 4 °C and delivered to the laboratory for assessing the dry weight proportion in relation to fresh weight. For calculating the proportion of dry tissue and humid tissue, the tissue was oven dried at 60 °C until reaching constant mass and then the dry weight was calculated by discounting the humidity in each sample.

### Determination of soil attributes

The physical and chemical attributes of the soil were determined using the procedures described in previous publications^63–65^. Attributes determined were pH, organic matter (OM), CaCO_3_, granulometric analysis (sand, clay and lime), exchangeable bases (Ca, Mg, Na, K), exchangeable acidity, cation-exchange capacity (CEC) and available P, S, B, Cu, Fe, Mn and Zn.

Granulometric analysis was performed via the Bouyoucos method using (NaPO_3_)_6_ + Na_2_CO_3_ as a dispersant. Soil pH (1:2.5 H_2_O) was measured using a potentiometer and organic carbon (OC) with the Walkley and Black method by titration, utilizing the factor of 1.7624 to calculate OM. Carbonates in soil were determined using a calcimeter and CEC and base cations (Ca^2+^, Mg^2+^, Na^+^, K^+^) were determined using extraction with 1.0 mol L^−1^ NH_4_OAc and then flame atomic absorption spectrophotometry (FAAS). To determine exchangeable acidity (Al^3+^, H^+^), Yuan’s method^66^ was used. Available P was extracted with the Olsen method (0.5 mol L^−1^ NaHCO_3_, pH 8.5) and determined using UV–VIS spectrophotometry. Available B was extracted with hot water and available S–SO_4_^2−^ with 500 mg L^−1^ P [Ca(H_2_PO_4_)·2H_2_O] + 2.0 mol L^−1^ CH_3_COOH, and determined using UV–VIS spectrophotometry. The selected microelements (Cu, Fe, Mn, Zn) were extracted with diethylenetriaminepentaacetic acid (DTPA) and then analyzed using FAAS. For soil chemical analysis, reference material AG-1 from SPC-Science was used to assure quality control.

### Determination of nutrient concentrations and uptake in “Bolaina” trees

After the tissues were dried (trunk section, branches and leaves), they were milled at 20 mesh and stored for plant analysis. Aliquots of 500 mg of each dried tissue were used to determine the macro (N, P, K, S, Ca, Mg) and micronutrients (B, Cu, Fe, Mn, Zn). For plant analysis, wet digestion methods were used (HNO_3_) and the procedures are detailed on Embrapa^65^. Determination of the elements can be summarized as follows: N was determined by the Kjeldahl method; P was determined by the ascorbic-molybdate color development method using UV–VIS spectrophotometry; and Ca, Mg, K, Cu, Fe, Mn and Zn were determined by FAAS using the Spectra 55B instrument from Varian. Concentrations are presented as the mean of three replicates from each repetition. In order to ensure analytical quality, a certified NIST material was used (Apple leaves).

For calculation of the macro- and micronutrient amounts, the following formula was used^67^:

$$Amount \left( {\text{mg per plant}} \right) = Concentration\, of\, element \,{*}\,Dry\, weight \,\left( {\text{g}} \right) {*}\,m$$

*Concentration* = results of chemical analysis of plant tissues (trunk, branches and leaves), in g kg^−1^ for macronutrients and mg kg^−1^ for micronutrients; *m* = 1 for macronutrients and 10^3^ for micronutrients.

### Allometric models

The nonlinear regression models used to build the allometric models are presented in Table 6. For the case of biometric models, with the exception of commercial height (CH) and diameter at breast height (DBH), all models were tested at a significant level of 0.05 for the best prediction algorithm. However, in the case of nutrient amounts, only Models 1, 2 and 7–10 were used.

**Table 6:** Nonlinear regression models used for the construction of allometric models, where diameter at breast height (DBH) and commercial height (CH) were used as independent variables (X).

Number	Model formula	Name and reference
1	$Y= a+b*DBH$	Linear model^68,69^
2	$Y= a+{b*DBH}^{0.5}+c*DBH$	Square root model^68,69^
3	$Y= a+b*log({DBH}^{2}*CH)$	Logarithmic model^68,69^
4	$Y= a{ ({DBH}^{2}*CH)}^{b}$	Modified power model^41,68,69^
5	$Y= a+b*{DBH}^{2}*CH$	Modified power model^41,68,69^
6	$Y= a{ *DBH}^{b}*{CH}^{c}$	Power model^68^
7	$Y= a *{DBH}^{b}$	Power model^41,68,69^
8	$Y= a+b*log(DBH)$	Logarithmic model^68,69^
9	$Y=\frac{a}{1+{e}^{b-c *DBH}}$	Logistic (Verhulst, 1838)^68,69^
10	$Y= a(1-{e}^{-c\left(DBH-b\right)})$	Mitscherlich model^70^

### Statistical analysis

All statistical analyses and graphics were calculated using R software, version 4.1.2^71^. For samples taken in the Huanuco region, owing to the proximity to the Ucayali region it was considered part of this region for all statistical analyses. Chemical and physical attributes of the soil were compared between the sampled regions by analysis of variance (ANOVA). In the case of biometric measurements and macro- and micronutrient amounts, linear and nonlinear regression models were constructed and compared using the Akaike information criterion (AIC) and the root-mean-square error (RMSE); the model with the lowest AIC and RMSE was selected as the best fit and the equation was presented for all the variables studied. Also, an *F*-test at a 0.05 significance level was performed in the selected models to verify the significance of the model.

### Supplementary Information

Supplementary Information.

## Data Availability

The datasets generated and/or analysed during the current study are not publicly available due the procedures established in contract with the funding institution but are available from the corresponding author on reasonable request.
