# Supplementary material for: Soil characteristics and allometric models for biometric characteristics and nutrient amounts for high yielding “Bolaina” (Guazuma crinita) trees
Source: Sci Rep. 2024 Jan 30;14:2444. doi: 10.1038/s41598-024-52790-1 (PMC10825134; doi:10.1038/s41598-024-52790-1)
Supplement: Supplementary file 1 — Supplementary Information. [file 41598_2024_52790_MOESM1_ESM.docx]

***Electronic Supplementary Information to*:**

**Soil characteristics and allometric models for biometric characteristics and nutrient amounts for high yielding “Bolaina” (Guazuma crinita) trees**

**Arevalo-Hernandez, C.O.^1,2,4^; Arévalo-Gardini, E. ^1,2^; Correa V., J. ^1^; Souza Júnior, J.O. ^3^; Neves L, J.C.^4^**

1. Department of Soils, Instituto de Cultivos Tropicales-ICT. Tarapoto-Perú.
2. Department of Agronomy, Universidad Nacional Autonoma de Alto Amazonas-UNAAA. Yurimaguas-Perú.
3. Department of Agricultural and Environmental Sciences. Universidade Estadual de Santa Cruz – UESC. Ilhéus, Brasil.
4. Department of Soils, Universidade Federal de Viçosa – UFV. Viçosa, Brasil.

* Correspondence author: [cesar.arevaloh@gmail.com](mailto:cesar.arevaloh@gmail.com)


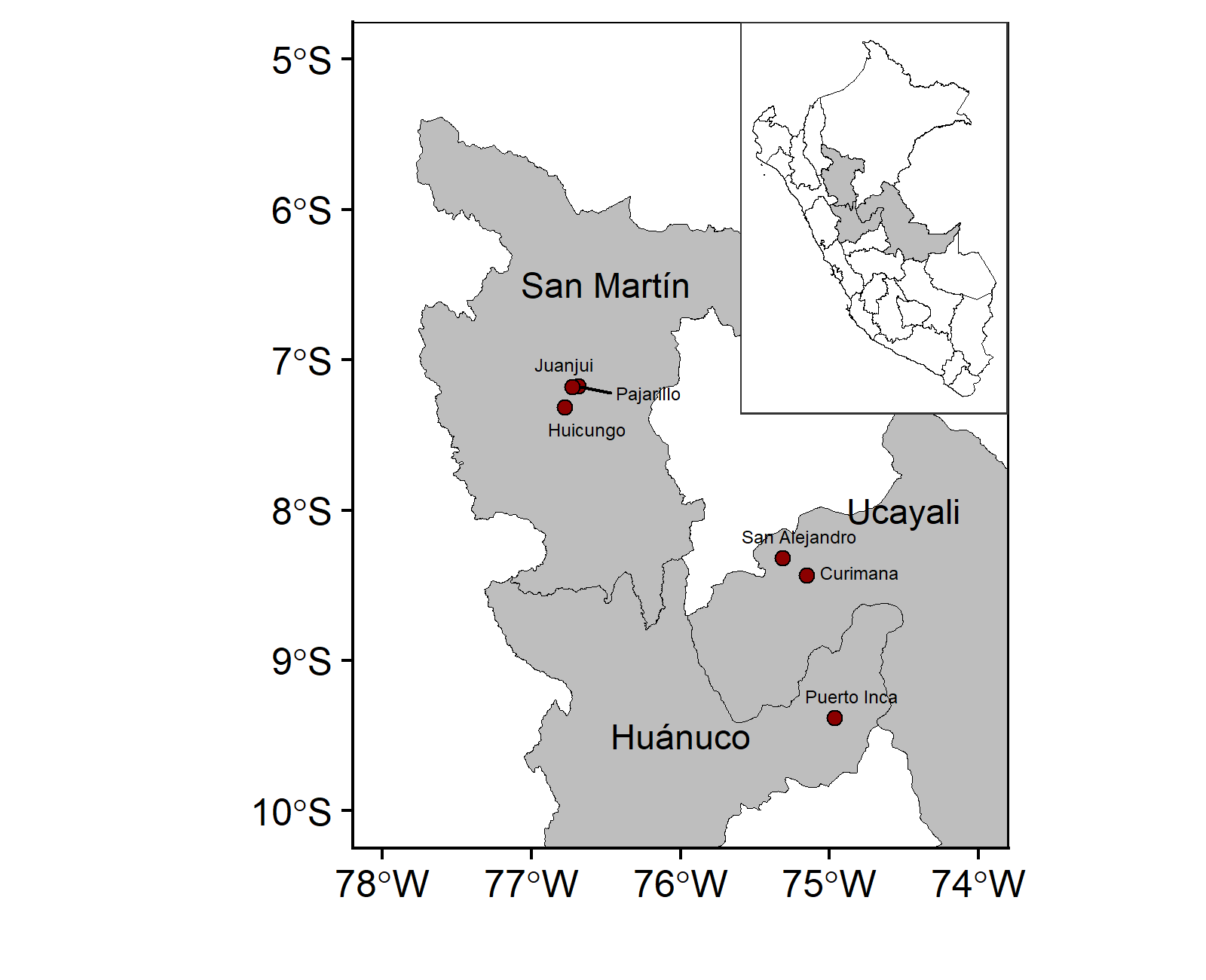


**Figure ESI-1.** Map of localization sites in San Martin (SM), Ucayali (UC) and Huánuco (HU) sampled in the study for soils, plant material and infield measurements

**Table ESI-1.** Mean ± standard deviation of diameter at breast height (DBH), commercial and total height, dry weight (trunk, leaves, branches and total) and wood volume per plant of Bolaina plants from different ages

| Age | DBH | Commercial height | Total height | Trunk dry weight | Leaves dry weight | Branches dry weight | Total dry weight | Wood volume |
| --- | --- | --- | --- | --- | --- | --- | --- | --- |
|  | cm | kg | m | kg | kg | kg | kg | m^3^ |
| 1 | 13.2 ± 1.3 | 5.7 ± 1.0 | 11.2 ± 1.3 | 22.4 ± 5.1 | 2.0 ± 0.8 | 15.0 ± 5.5 | 39.3 ± 8.7 | 0.79 ± 0.30 |
| 3 | 16.9 ± 3.0 | 11.3 ± 3.8 | 18.1 ± 6.4 | 65.4 ± 39.1 | 6.2 ± 4.1 | 10.5 ± 4.8 | 82.1 ± 39.8 | 2.83 ± 1.87 |
| 5 | 19.7 ± 1.4 | 15.4 ± 2.3 | 23.4 ± 3.7 | 99.4 ± 19.0 | 5.0 ± 4.1 | 15.8 ± 8.5 | 120.2 ± 19.1 | 4.68 ± 1.00 |
| 7 | 21.9 ± 1.8 | 16.4 ± 2.0 | 24.8 ± 3.0 | 130.1 ± 23.1 | 4.3 ± 4.3 | 27.8 ± 17.6 | 162.2 ± 29.3 | 6.13 ± 1.45 |
| 10 | 31.4 ± 3.7 | 13.5 ± 3.2 | 22.1 ± 5.2 | 185.9 ± 41.9 | 22.3 ± 8.5 | 24.7 ± 19.6 | 232.9 ± 47.7 | 10.07 ± 0.84 |

**Table ESI-2.** Mean ± standard deviation concentration of nutrients and nutrient uptake of N, P, K, Ca, Mg, S, B, Cu, Fe, Mn and Zn of Bolaina plants from different ages

| **Age** | **N** | | | **P** | | | **K** | | | **Ca** | | | **Mg** | | | **S** | | | **B** | | | **Cu** | | | **Fe** | | | **Mn** | | | **Zn** | | |
| --- | --- | --- | --- | --- | --- | --- | --- | --- | --- | --- | --- | --- | --- | --- | --- | --- | --- | --- | --- | --- | --- | --- | --- | --- | --- | --- | --- | --- | --- | --- | --- | --- | --- |
|  | Leaves | Branches | Trunk | Leaves | Branches | Trunk | Leaves | Branches | Trunk | Leaves | Branches | Trunk | Leaves | Branches | Trunk | Leaves | Branches | Trunk | Leaves | Branches | Trunk | Leaves | Branches | Trunk | Leaves | Branches | Trunk | Leaves | Branches | Trunk | Leaves | Branches | Trunk |
|  | **NUTRIENT CONCENTRATION** | | | | | | | | | | | | | | | | | | | | | | | | | | | | | | | | |
|  | **g kg^-1^** | | | | | | | | | | | | | | | | | | **mg kg^-1^** | | | | | | | | | | | | | | |
| 1 | 26.8±4.8 | 7.5±2.7 | 4.6±0.4 | 2.9±0.1 | 0.9±0.1 | 0.7±0.1 | 13.6±4.3 | 10.2±2.6 | 6.2±0.9 | 23.6±4.1 | 15±3.3 | 3.3±1.3 | 2.8±0.4 | 1.9±0.3 | 0.6±0 | 1.5±0.2 | 0.8±0.1 | 0.7±0.1 | 14.0 ± 2.6 | 6.5 ± 1.0 | 6.1 ± 0.9 | 9.4 ± 1.6 | 11.2 ± 1.9 | 4.7 ± 1.1 | 78.3 ± 22.7 | 38.0 ± 11.2 | 42.99 ± 41.11 | 41.2 ± 11.7 | 13.0 ± 4.5 | 2.5 ± 1.2 | 24.5 ± 5.3 | 19.8 ± 3.3 | 4.2 ± 1.5 |
| 3 | 24.6±2.7 | 5.7±1.8 | 5.8±1.5 | 3.2±0.6 | 1±0.2 | 0.6±0 | 13.2±2.7 | 8.3±4.2 | 4.9±1 | 19.2±2.7 | 5.9±2.4 | 3.5±1.5 | 4±1.5 | 1±0.4 | 0.6±0.2 | 1.3±0.2 | 0.9±0.4 | 0.7±0.2 | 12.5 ± 1.6 | 8.5 ± 3.5 | 9.3 ± 4.0 | 9.3 ± 2.5 | 6.8 ± 4.3 | 3.1 ± 1.7 | 94.1 ± 43.4 | 41.4 ± 23.6 | 33.76 ± 15.35 | 84.0 ± 43.4 | 11.1 ± 6.6 | 4.8 ± 3.0 | 22.9 ± 6.7 | 10.4 ± 4.6 | 4.3 ± 3.0 |
| 5 | 27.7±2.4 | 5.9±2.3 | 4.1±0.6 | 3.3±0.6 | 1.1±0.2 | 0.7±0.1 | 12.7±1.2 | 10.3±4.7 | 3.8±0.7 | 22.4±1.7 | 11±8.9 | 3.8±0.4 | 2.7±0.6 | 1.2±0.6 | 0.4±0 | 1.3±0.4 | 0.8±0.3 | 1±0.8 | 11.6 ± 2.6 | 8.3 ± 4.6 | 8.9 ± 4.8 | 11.4 ± 2.7 | 11.1 ± 8.2 | 2.2 ± 0.7 | 94.1 ± 42.7 | 30.0 ± 9.0 | 39.88 ± 31.45 | 61.0 ± 39.5 | 14.1 ± 7.6 | 2.4 ± 1.2 | 21.2 ± 11.9 | 14.4 ± 7.9 | 2.5 ± 2.0 |
| 7 | 24.6±3.2 | 5.8±2.1 | 4.9±2.3 | 3±0.2 | 0.9±0.1 | 0.6±0 | 9.3±2.2 | 8.1±4.9 | 3.9±1.5 | 18.6±7 | 13.5±7.2 | 5.4±3.4 | 3±1.4 | 1.1±0.7 | 0.5±0.2 | 1.3±0.6 | 0.8±0.1 | 0.8±0.4 | 12.7 ± 3.8 | 12.4 ± 7.4 | 13.8 ± 6.1 | 11.1 ± 3.9 | 9.1 ± 4.4 | 2.3 ± 1.1 | 66.5 ± 10.6 | 40.1 ± 10.1 | 30.0 ± 9.2 | 35.6 ± 8.9 | 8.9 ± 5.5 | 4.7 ± 2.8 | 22.1 ± 9.1 | 12.8 ± 6.6 | 3.4 ± 1.7 |
| 10 | 20.8±0.5 | 5.8±0.5 | 4±1 | 2.7±0.1 | 0.8±0 | 0.6±0 | 7.4±0.4 | 3.5±0.1 | 2.6±1.2 | 29.1±5.2 | 12.8±5.2 | 8.6±1 | 1.8±0 | 0.6±0.1 | 0.2±0.1 | 1±0.4 | 0.6±0.1 | 0.5±0.1 | 8.7 ± 4.1 | 9.1 ± 9.3 | 7.1 ± 4.5 | 7.0 ± 0.6 | 8.3 ± 5.5 | 1.7 ± 0.6 | 66.7 ± 22.8 | 112.4 ± 233.1 | 45.5 ± 37.9 | 26.9 ± 2.6 | 5.0 ± 1.3 | 1.8 ± 0.4 | 18.5 ± 3.9 | 6.9 ± 5.6 | 2.3 ± 1.3 |
|  | **NUTRIENT UPTAKE** | | | | | | | | | | | | | | | | | | | | | | | | | | | | | | | | |
|  | **g per plant** | | | | | | | | | | | | | | | | | | **mg per plant** | | | | | | | | | | | | | | |
| 1 | 59.2 ± 10.5 | 122.3 ± 67.1 | 104.0 ± 32.6 | 6.4 ± 1.0 | 15.3 ± 7.1 | 15.0 ± 4.0 | 29.5 ± 6.8 | 159.8 ± 73.1 | 137.8 ± 33.6 | 53.4 ± 13.8 | 246.7 ± 123.1 | 76.4 ± 35.1 | 6.3 ± 1.6 | 30.6 ± 14.2 | 12.2 ± 2.0 | 3.5 ± 1.1 | 13.3 ± 5.8 | 15.8 ± 4.0 | 32.4 ± 10.8 | 100.5 ± 30.6 | 135.7 ± 32.0 | 21.0 ± 5.00 | 182.9 ± 94.3 | 104.4 ± 31.3 | 179.9 ± 77.0 | 559.0 ± 141.2 | 912.9 ± 777.2 | 90.8 ± 25.9 | 212.3 ± 115.3 | 52.6 ± 20.2 | 55.4 ± 17.3 | 311.6 ± 112.0 | 92.8 ± 36.0 |
| 3 | 147.2 ± 109.9 | 51.3 ± 18.7 | 386.2 ± 354.3 | 21.0 ± 18.0 | 9.3 ± 3.0 | 38.7 ± 24.9 | 86.6 ± 73.1 | 68.5 ± 19.1 | 286.0 ± 168.5 | 110.4 ± 72.2 | 63.3 ± 49.2 | 203.9 ± 136.2 | 21.2 ± 15.6 | 10.0 ± 7.1 | 30.2 ± 13.2 | 7.2 ± 4.7 | 9.5 ± 7.8 | 35.0 ± 11.9 | 75.3 ± 56.0 | 94.1 ± 74.5 | 452.9 ± 148.2 | 62.8 ± 53.7 | 53.0 ± 23.0 | 197.2 ± 208.2 | 469.4 ± 326.3 | 323.7 ± 88.0 | 2,170.3 ± 1,773.0 | 370.2 ± 174.0 | 121.9 ± 120.9 | 260.1 ± 192.9 | 125.4 ± 98.6 | 91.1 ± 45.1 | 227.5 ± 184.4 |
| 5 | 174.5 ± 151.2 | 112.2 ± 73.6 | 403.4 ± 105.5 | 21.8 ± 19.9 | 18.9 ± 8.9 | 69.9 ± 22.4 | 74.7 ± 62.2 | 196.8 ± 152.6 | 385.2 ± 131.6 | 129.5 ± 102.5 | 226.8 ± 235.6 | 376.5 ± 94.8 | 18.2 ± 16.6 | 24.3 ± 19.6 | 38.2 ± 9.1 | 6.4 ± 4.2 | 16.1 ± 10.7 | 92.6 ± 56.7 | 60.1 ± 42.0 | 157.4 ± 119.5 | 860.7 ± 513.8 | 68.5 ± 59.5 | 225.4 ± 224.1 | 211.8 ± 62.8 | 622.1 ± 730.3 | 509.2 ± 255.3 | 3,889.8 ± 2,866.2 | 219.2 ± 67.6 | 290.6 ± 203.2 | 243.0 ± 128.3 | 91.5 ± 86.5 | 274.6 ± 223.8 | 265.6 ± 258.7 |
| 7 | 115.8 ± 124.5 | 158.4 ± 110.8 | 610.3 ± 200.2 | 13.0 ± 13.3 | 23.6 ± 14.1 | 81.4 ± 12.9 | 42.6 ± 48.2 | 241.3 ± 198.5 | 516.2 ± 219.6 | 69.2 ± 80.9 | 310.1 ± 159.7 | 705.6 ± 485.5 | 9.9 ± 11.8 | 31.8 ± 22.4 | 62.8 ± 30.8 | 3.6 ± 2.4 | 21.3 ± 13.4 | 94.9 ± 35.0 | 62.3 ± 67.8 | 422.0 ± 418.0 | 1,869.2 ± 969.3 | 56.6 ± 63.1 | 217.6 ± 96.5 | 305.2 ± 197.4 | 277.5 ± 274.4 | 1,498.9 ± 1,944.4 | 6,041.1 ± 5,497.5 | 134.3 ± 131.5 | 280.7 ± 248.9 | 614.3 ± 371.2 | 117.3 ± 133.4 | 300.8 ± 151.2 | 445.3 ± 274.1 |
| 10 | 474.2 ± 260.4 | 178.8 ± 112.6 | 715.4 ± 45.2 | 62.2 ± 34.7 | 25.9 ± 18.1 | 117.3 ± 32.2 | 171.1 ± 98.9 | 108.7 ± 73.1 | 467.7 ± 181.6 | 691.7 ± 468.0 | 350.0 ± 117.4 | 1,571.8 ± 146.5 | 40.7 ± 22.0 | 17.3 ± 10.2 | 35.3 ± 10.3 | 21.3 ± 3.2 | 17.0 ± 8.8 | 90.7 ± 17.9 | 172.6 ± 11.2 | 187.1 ± 95.7 | 1,389.7 ± 984.3 | 162.8 ± 98.2 | 322.1 ± 354.5 | 296.8 ± 73.8 | 1,376.5 ± 283.9 | 1,159.4 ± 557.9 | 5,343.5 ± 706.7 | 625.2 ± 382.5 | 143.1 ± 67.7 | 317.7 ± 28.1 | 443.8 ± 310.7 | 278.4 ± 326.4 | 413.8 ± 195.9 |

**Table ESI-3.** Allometric models evaluation with Akaike Information Criterion (AIC) and Root Mean Squared Error (RMSE) for macronutrient (N, P, K, Ca, Mg, S) amounts of trunk and total in g per plant of “bolaina” (*Guazuma crinita*) trees from 1 to 10 years in the sampled areas in San Martin and Ucayali department

| **Models** | **N** | | | | **P** | | | | **K** | | | | **Ca** | | | | **Mg** | | | | **S** | | | |
| --- | --- | --- | --- | --- | --- | --- | --- | --- | --- | --- | --- | --- | --- | --- | --- | --- | --- | --- | --- | --- | --- | --- | --- | --- |
|  | Trunk | | Total | | Trunk | | Total | | Trunk | | Total | | Trunk | | Total | | Trunk | | Total | | Trunk | | Total | |
|  | AIC | RMSE | AIC | RMSE | AIC | RMSE | AIC | RMSE | AIC | RMSE | AIC | RMSE | AIC | RMSE | AIC | RMSE | AIC | RMSE | AIC | RMSE | AIC | RMSE | AIC | RMSE |
| **1*** | 50.05 | 0.48 | 25.36 | 0.33 | 37.13 | 0.39 | 11.04 | 0.26 | 36.22 | 0.39 | 21.56 | 0.31 | 53.20 | 0.51 | **25.37** | **0.33** | 48.67 | 0.47 | 20.79 | 0.31 | 61.29 | 0.57 | 41.20 | 0.43 |
| **2** | **21.43** | **0.30** | **19.04** | **0.29** | -9.33 | 0.18 | 0.94 | 0.22 | **17.25** | **0.28** | **14.90** | **0.27** | **39.97** | **0.40** | 26.69 | 0.33 | **27.75** | **0.33** | **16.32** | **0.28** | 46.10 | 0.44 | 36.55 | 0.38 |
| **7** | 37.76 | 0.40 | 19.87 | 0.30 | 18.47 | 0.29 | 2.50 | 0.23 | 27.22 | 0.34 | 17.81 | 0.29 | 41.75 | 0.42 | 29.07 | 0.35 | 41.34 | 0.42 | 17.90 | 0.29 | 54.53 | 0.52 | 37.58 | 0.40 |
| **8** | 42.90 | 0.43 | 21.41 | 0.31 | 30.45 | 0.35 | 6.30 | 0.24 | 29.90 | 0.35 | 18.45 | 0.30 | 48.27 | 0.47 | 27.43 | 0.34 | 44.70 | 0.44 | 18.56 | 0.30 | 57.95 | 0.54 | 39.03 | 0.41 |
| 9 | 21.56 | 0.30 | 19.06 | 0.29 | **-11.61** | **0.18** | **0.56** | **0.21** | 17.48 | 0.28 | 16.47 | 0.28 | 40.44 | 0.40 | 26.67 | 0.38 | 29.13 | 0.34 | 17.41 | 0.28 | **46.08** | **0.44** | **36.39** | **0.38** |
| 10 | 23.34 | 0.31 | 19.31 | 0.29 | -6.64 | 0.19 | 0.92 | 0.22 | 18.41 | 0.28 | 16.93 | 0.28 | 40.81 | 0.40 | 26.93 | 0.38 | 30.18 | 0.34 | 17.58 | 0.28 | 46.69 | 0.44 | 36.34 | 0.38 |

*The selected models were significant by F-test at 0.05. Values in bold represent the best model, with the lower AIC (Akaike Information Criterion) and RMSE (Root Mean Squared Error)

**Table ESI-4.** Allometric models evaluation with Akaike Information Criterion (AIC) and Root Mean Squared Error (RMSE) for micronutrient (B, Cu, Fe, Mn, Zn) amountamount of trunk and total in g per plant of “bolaina” (*Guazuma crinita*) trees from 1 to 10 years in the sampled areas in San Martin and Ucayali department

| **Models** | **B** | | | | **Cu** | | | | **Fe** | | | | **Mn** | | | | **Zn** | | | |
| --- | --- | --- | --- | --- | --- | --- | --- | --- | --- | --- | --- | --- | --- | --- | --- | --- | --- | --- | --- | --- |
|  | Trunk | | Total | | Trunk | | Total | | Trunk | | Total | | Trunk | | Total | | Trunk | | Total | |
|  | AIC | RMSE | AIC | RMSE | AIC | RMSE | AIC | RMSE | AIC | RMSE | AIC | RMSE | AIC | RMSE | AIC | RMSE | AIC | RMSE | AIC | RMSE |
| **1** | 77.30 | 0.74 | 62.94 | 0.61 | 50.02 | 0.48 | 37.23 | 0.40 | 73.53 | 0.70 | 54.76 | 0.53 | 79.71 | 0.77 | 42.59 | 0.44 | 64.86 | 0.61 | 37.22 | 0.40 |
| **2** | 69.11 | 0.63 | 59.72 | 0.56 | **40.80** | **0.40** | **37.38** | **0.39** | **61.06** | **0.55** | **51.44** | **0.49** | **69.87** | **0.64** | **41.66** | **0.42** | 64.07 | 0.58 | 38.88 | 0.40 |
| **7** | 72.24 | 0.68 | 60.13 | 0.58 | 45.02 | 0.45 | 36.87 | 0.39 | 66.98 | 0.63 | 51.46 | 0.50 | 75.19 | 0.71 | 41.38 | 0.43 | **62.75** | **0.59** | **36.94** | **0.40** |
| **8** | 74.15 | 0.70 | 61.01 | 0.59 | 46.43 | 0.46 | 38.08 | 0.39 | 69.12 | 0.65 | 52.30 | 0.51 | 76.96 | 0.73 | 41.57 | 0.43 | 63.33 | 0.59 | 36.95 | 0.40 |
| 9 | **68.93** | **0.63** | **59.10** | **0.56** | 42.11 | 0.41 | 37.25 | 0.39 | 62.18 | 0.56 | 51.59 | 0.49 | 71.47 | 0.65 | 41.59 | 0.49 | 64.03 | 0.58 | 38.74 | 0.40 |
| 10 | 69.02 | 0.63 | 60.13 | 0.56 | 42.58 | 0.42 | 37.44 | 0.39 | 62.79 | 0.57 | 51.86 | 0.49 | 71.79 | 0.66 | 42.71 | 0.42 | 64.10 | 0.58 | 38.80 | 0.40 |

*The selected models were significant by F-test at 0.05. Values in bold represent the best model, with the lower AIC (Akaike Information Criterion) and RMSE (Root Mean Squared Error)
